# Correlation between semiautomated magnetic resonance imaging volumetry of the cingulate gyrus and interictal epileptiform discharge lateralization in dogs with idiopathic epilepsy

**DOI:** 10.1111/jvim.17178

**Published:** 2024-08-27

**Authors:** Aleksandra Banasik, Marcin Wrzosek, Paulina Drobot, Karolina Owsińska‐Schmidt, Laura Brewińska, Anna Zimny, Przemysław Podgórski

**Affiliations:** ^1^ Department of Internal Medicine and Clinic of Diseases of Horses, Dogs and Cats Wrocław University of Environmental and Life Sciences Wrocław Poland; ^2^ NeuroTeam Specialist Veterinary Clinic Wrocław Poland; ^3^ Department of General and Interventional Radiology and Neuroradiology Wrocław Medical University Wrocław Poland

**Keywords:** brain imaging, limbic system, retrospective clinical study, veterinary medicine

## Abstract

**Background:**

Brain imaging suggests the involvement of the limbic system, particularly the cingulate gyrus (GC), in dogs with idiopathic epilepsy (IE).

**Hypothesis:**

A correlation exists between the side of interictal epileptiform discharges (IEDs) and the volume of the ipsilateral GC (GCe) in dogs with IE.

**Animals:**

Dogs admitted to the neurological consultation (32 with epileptic seizures and 13 control) were included.

**Methods:**

This retrospective, blinded study followed the International Veterinary Epilepsy Task Force recommendations for diagnosing IE at the Tier III confidence level. The IE group included 18 and 14 dogs with IEDs in the left and right hemispheres, respectively (median age: 36 months, median weight: 19.5 kg), whereas the control group included 13 dogs (median age: 32 months, median weight: 20 kg). Whole‐brain and GC‐volumetric assessments were performed by a semiautomated method.

**Results:**

In the control group, the volume of the GC was: left, from 743.63 to 1001.61 mm^3^, right, from 789.35 to 1015.86 mm^3^. In the study group, the volume of the GC was: left, from 720.88 to 1054.9 mm^3^ and right, from 566.29 to 987.77 mm^3^. In dogs with IE, GCe volume was significantly lower than the mean volume of the GC in the control group relative to total intracranial volume (TIV; *P* = .00044).

**Conclusions and Clinical Importance:**

Alterations in the volume of the GC provide insights into structural changes during IE. The use of semiautomatic volumetry provides an advantage by reducing the potential for human error.

**Abbreviations**
CD: cortical dysgenesis
CSF: cerebrospinal fluid
DRE: drug resistant epilepsy
ED: epileptiform discharge
EEG: electroencephalography
FCD: focal cortical dysgenesis
GC: cingulate gyrus
GSW: generalized spike‐and‐wave
IE: idiopathic epilepsy
IGE: idiopathic generalized epilepsy
IVETF: International Veterinary Epilepsy Task Force
MR: magnetic resonance
MRI: magnetic resonance imaging
SITs: superimposed transients
TIV: total intracranial volume
VBM: volume‐based morphometry
VOI: volume of interest

## 1 INTRODUCTION

Recent research on epilepsy, particularly on idiopathic epilepsy (IE), is extensive, with a diagnosis rate of 0.6% to 0.75% in the canine population.^1^, ^2^, ^3^, ^4^, ^5^, ^6^, ^7^, ^8^ According to the International Veterinary Epilepsy Task Force (IVETF), IE is classified based on etiology into genetic epilepsy, suspected genetic epilepsy, and epilepsy of unknown etiology, and based on phenotypic manifestations into focal epileptic seizure, generalized epileptic seizure, and focal epileptic seizure evolving into generalized seizures. Idiopathic epilepsy refers to epilepsy wherein the underlying cause is unknown and there is no indication for structural epilepsy.^9^, ^10^ Approximately 30% of focal epileptogenic lesions are missed on standard magnetic resonance imaging (MRI) in human medicine, and advanced neuroimaging modalities are required in these cases.^11^

Electroencephalography (EEG) is a diagnostic tool increasingly used for epilepsy in veterinary clinical centers worldwide.^12^, ^13^, ^14^ It allows the assessment of cortical activity and identification of abnormalities by observing visible epileptiform discharges (EDs) or interictal epileptiform discharges (IEDs) and their locations.^15^ According to the IVETF, ED identification by EEG enables the diagnosis of IE with the highest level of confidence (Tier III).^16^

Research based on volume‐based morphometry (VBM) in human medicine suggests that patients with idiopathic generalized epilepsy (IGE) such as exhibit structural abnormalities in the cingulate gyrus (GC), mainly in the cranial and caudal portions.^17^, ^18^, ^19^ There are structural changes in the brain (including the limbic system) during epileptic activity, suggesting epilepsy as a network disease.^20^, ^21^

The GC, situated in the medial aspect of the cerebral hemispheres, is a component of the limbic system, interconnected with the parahippocampal gyrus, neocortex, and rostral thalamus. It is considered a crucial structure in the propagation of epileptic discharges because of its diffused connections.^22^, ^23^ In veterinary medicine, the GC, hippocampus, and piriform lobe are identified as the areas affected by postictal lesions.^24^

Magnetic resonance (MR) automated brain volumetry is a tool developed in human medicine.^25^, ^26^ Unfortunately, owing to the diversity of species and a lack of software and population atlases, automating and analyzing certain small structures in dog brains has not been possible. Until now, only manual brain volumetry, with a lower sensitivity than automated methods, has been possible in veterinary medicine. Current veterinary tools allow the segmentation of MR images and volume calculations of the whole brain or selected regions. Brain atlases are also available that allow semiautomatic volumetric analysis of selected brain structures, resulting in more reliable results.^27^, ^28^

According to the literature, 53% to 63% of seizure cases in veterinary medicine are annually described as generalized tonic‐clonic seizures.^1^, ^29^ Given the limited understanding of the interplay between brain structure and epilepsy in dogs, alongside the demand for more effective diagnostic tools in veterinary neurology, this study aimed to establish a correlation between semiautomated volumetry of the GC and the localization of IEDs in dogs with IE.

## 2 MATERIALS AND METHODS

### 2.1 Case selection

This retrospective clinical study was conducted on dogs referred for neurological consultation and EEG at the Clinic for Horses, Dogs, and Cats at the Department of Internal Medicine at the University of Environmental and Life Sciences in Wroclaw, Poland, between 2015 and 2021. Magnetic resonance imaging studies were performed at the Centre for Experimental Diagnostics and Biomedical Innovations of the Faculty of Veterinary Medicine, Wroclaw University of Environmental and Life Sciences, Poland. Owners signed consent before EEG and MRI examinations. According to Polish law, ethical committee approval was not mandated for this study (Experiments on Animals Act from January 15, 2015, *Journal of Laws of the Republic of Poland* from 2015, item. 266).

#### 2.1.1 Inclusion criteria

The inclusion criteria for the study group were as follows: a mesaticephalic skull,^30^, ^31^ age between 6 months and 6 years at seizure onset, history of primary generalized tonic‐clonic seizures (from anamnesis and video recordings), unremarkable interictal clinical and neurological examination results, unremarkable blood analysis results (complete blood count, biochemistry including bile acids and ammonia), unremarkable findings on conventional MRI examination performed according to the IVETF protocol, and unremarkable cerebrospinal fluid (CSF) analysis findings.

The inclusion criteria for the control group were as follows: a mesaticephalic skull, no history of seizures or any other forebrain disease, normal clinical and neurological examination results, normal blood parameters, and normal brain MRI findings.

#### 2.1.2 Exclusion criteria

The exclusion criterion was a history of cluster seizures or status epilepticus.

### 2.2 EEG recording

The 30‐minute EEG study adhered to a previously outlined protocol.^32^ All dogs received sedation via intramuscular injections of medetomidine (Narcostart, Livisto, Gdynia, Poland). The drug was injected into the right triceps muscle at a dose of 20 μg/kg. Recordings were performed before the start of general anesthesia for MRI and CSF examinations.

Subdermal wire electrodes (Ives EEG Solutions, Newbury Port, MA, USA) were used for recordings. Dogs were positioned in the sternal position to facilitate videometry, which recorded any potential movements during EEG. Recordings were conducted by an EEG machine (Nikon Kohden, Rosbach, Germany) with the following settings: sensitivity 70 μV/cm, bandpass filter set to 0.5 to 30 Hz, time constant 0.3, and an inserted 50‐Hz notch filter. Each recording was performed by a 10‐channel referential montage (F3, F4, C3, C4, T3, T4, O1, O2‐Ref., with the reference electrode placed on the frontal bone and the ground electrode inserted in the neck) as well as a standard bipolar montage (F3‐C3, C3‐T3, T3‐O1, F4‐C4, C4‐T4, T4‐O2). The ECG‐Ref. electrode was placed SC at the level of the left fifth intercostal space near the chondrocostal junction.

Light stimulation was administered during the 10th minute of EEG recording with a stroboscope lamp. The initial stimulation frequency commenced at 0.5 Hz and gradually increased to 60 Hz, before steadily returning to the baseline over a 5‐minute period, as previously reported.^13^

A veterinary neurologist blindly analyzed the recordings by both mono‐ and bipolar montages with videometric control.

Visual analysis reviewed pathological IEDs and physiologically superimposed transients (SITs). The currently accepted nomenclature was used to identify IEDs.^15^, ^33^ Interictal epileptiform discharge localization was defined based on the highest amplitude of discharge in a reference montage and the reversed polarity in a bipolar montage recording. Interictal epileptiform discharges were differentiated from physiologic SITs including occipital or frontal intermittent rhythmic, delta activity, sleep spindles, K‐complexes, as well as artifacts such as muscle activity, ocular, and muscular movements.

### 2.3 Magnetic resonance imaging

After EEG recording, the dogs underwent MRI scans. Anesthesia was induced by propofol (3‐5 mg/kg; Propofol Lipuro, 10 mg/mL, B Braun, Melsungen AG), and maintenance was achieved with inhaled isoflurane in oxygen (1.5‐2% vol.). Magnetic resonance imaging was conducted in dorsal recumbency by a 1.5 T scanner (Philips, Ingenia, Philips Healthcare, Eindhoven, Holland). A 1.5 T head‐neck coil (Philips) was used in this study. The IVETF epilepsy‐specific MRI protocol was used to scan the brains of the dogs by 3‐dimensional (3D) T1 (TR [repetition time] 25 ms/TE [echo time] 6.2 ms, FOV [field of view] 180 × 180 mm, matrix size 256 × 256, voxel 0.75 × 0.75 mm, slice thickness 0.7 mm, slice gap = 0 mm [isotropic acquisition and reconstruction]) by Turbo Field Echo (TFE) technique; T2‐weighted (T2W) sequence (TR 8042 ms/TE 100 ms, FOV 120 × 100 mm, matrix size 268 × 171 mm, voxel 0.45 × 0.569 mm, slice thickness 2 mm, slice gap 1 mm), by Turbo Spin Echo (TSE) technique; fluid‐attenuated inversion recovery (FLAIR; TR 9000 ms/TE 140 ms/TI 2450 ms), by TSE technique; T2* sequence by Fast Field Echo (FFE) technique, and 3D T1 sequence with a contrast agent. Transverse plane T2W images were acquired for all dogs.^16^, ^34^

Magnetic resonance imaging scans were assessed by the authors and validated by a board‐certified neurologist with a Diploma from the European College of Veterinary Neurology. Visual inspection of MR images revealed no structural changes.

### 2.4 MR volumetric analysis

The initial phase of the analysis involved utilizing both manual and semiautomated techniques for brain segmentation. This segmentation was conducted by MRIcron software v1.0.20190902 (NITRC NeuroImaging Tools and Resources Collaboratory, NIH Grant number: 1R24EB029173 led by the University of Massachusetts Medical School in Worcester, United States). Multiple manual 3D volume of interest (VOI) selections were performed based on background intensity to delineate regions of interest, including the brain, cerebellum, and spinal cord. Parameters such as VOI radius, erosion/dilatation cycles, and differences in intensity from the origin and edge were meticulously adjusted to ensure accurate isolation of the desired tissues while excluding extraneous structures. Skull stripping was unnecessary following this procedure because the extracted brain and cerebellum were already free of the skull.

Subsequently, the extracted brain underwent coregistration with a stereotactic Cortical Atlas of the Domestic Canine Brain^27^ by Statistical Parametric Mapping 12 (SPM12) (Functional Imaging Laboratory (FIL), the Wellcome Trust Centre for NeuroImaging [WTCN], in the Institute of Neurology and the University College London [UCL], United Kingdom). This involved aligning the data to the atlas space, followed by segmentation into gray matter, white matter, and CSF. The affine registration functionality of 3D Slicer was utilized to precisely align each dataset with the atlas, ensuring spatial accuracy.^35^

**FIGURE 1:**
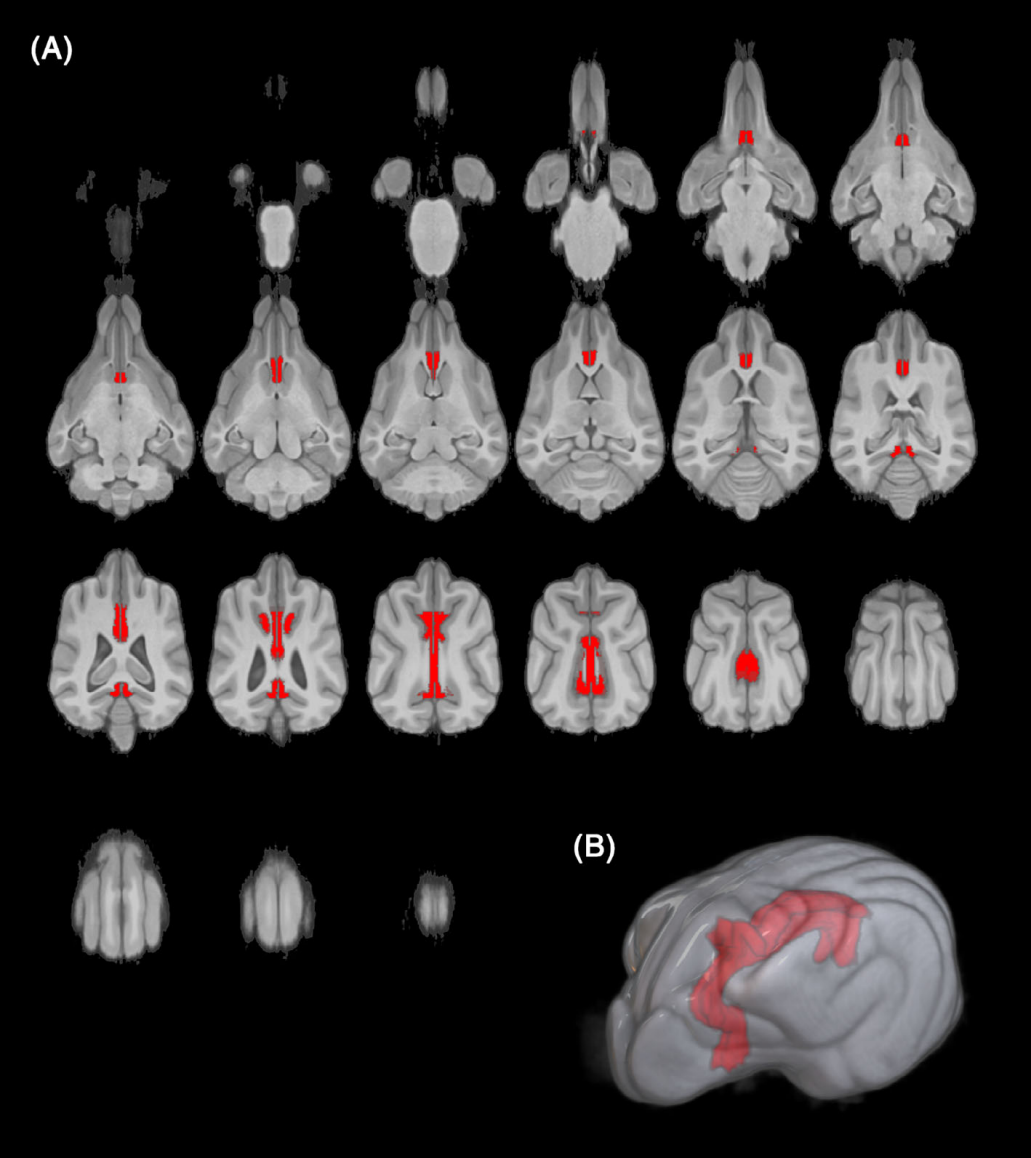
Visualization of the matched cingulate gyrus on magnetic resonance imaging in T1‐weighted sequence in 3‐dimensional planes (A) and 3‐dimensional reconstruction of the cingulate gyrus (B).

**FIGURE 2:**
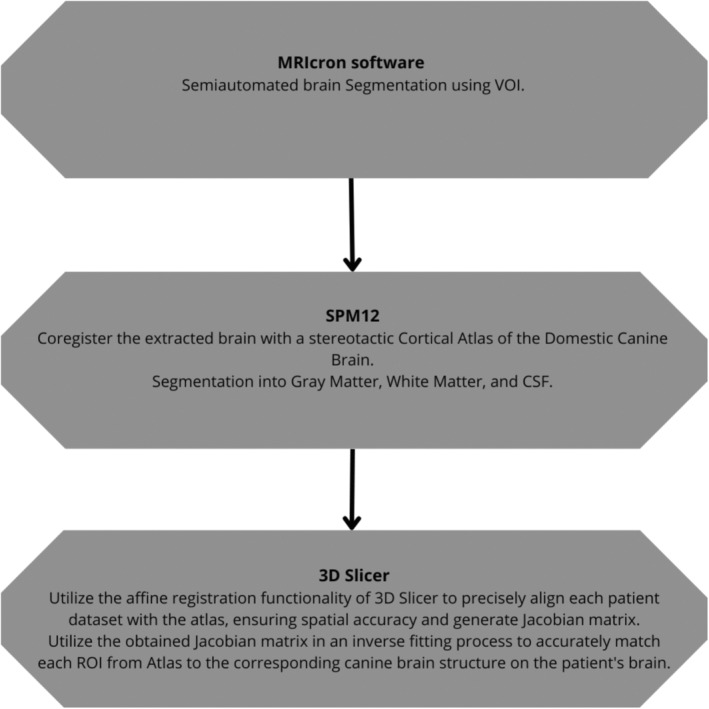
A scheme describing the application of the various stages of segmentation.

The obtained Jacobian matrix was then utilized in an inverse fitting process to accurately match each region of interest to the corresponding canine brain structure. Visualization of the matched temporal lobes on MRI in a T1W sequence in 3D planes and a 3D reconstruction of the GC is depicted in Figure 1. Notably, the volume of the fitted GC VOI closely corresponded to the individual subject's GC volume, validating the accuracy of the segmentation and registration processes. A scheme describing the application of the various stages of segmentation is shown in Figure 2.

### 2.5 Data analysis

Data are presented as median with first and third quartiles (Table 1). Normal distribution was verified by the Shapiro‐Wilk test. Variables with normal distribution were compared between the groups by Student's *t* test, and those with nonnormal distribution were compared by the Mann‐Whitney *U* test. Pearson's coefficient was used to assess the correlations.

**TABLE 1:** The baseline characteristics of the groups.

	Study group			Control group			*P*
	Median	q1	q3	Median	q1	q3	
Weight (kg)	19.5	14.2	26.5	20.0	18.0	23.0	.759
Age (months)	36.0	24.0	52.5	32.0	25.0	66.0	.841
White matter volume (mm^3^)	30 332.0	26 499.0	33 320.0	31 317.0	27 371.0	33 548.0	.710
Gray matter volume (mm^3^)	50 521.0	45 463.0	57 395.0	50 859.0	46 705.0	54 328.0	.692
CSF volume (mm^3^)	22 444.0	20 146.0	25 110.0	21 234.0	20 415.0	21 555.0	.356
White matter_CSF	1.4	1.3	1.4	1.5	1.3	1.5	.138*
Gray matter_CSF	37 171.0	32 699.0	40 604.0	34 215.0	31 812.0	36 898.0	.191*
Gray matter_White matter_CSF	3.6	3.3	3.8	3.8	3.7	3.9	.125*
Gray matter_White matter	0.6	0.6	0.7	0.6	0.6	0.6	.414
Cingulate L (mm^3^)	858	788	914	889	841	912	.374
Cingulate R (mm^3^)	793	747	880	885	805	931	.0523

* denotes statistically significant values.

In the first stage of the study, gray matter, white matter, CSF, and total intracranial volumes (TIV) were compared between the IE and control groups.

Subsequently, for further analyses, data were organized by creating 2 variables: the ratio of GC volume on the side of the epileptogenic discharges to total intracranial volume (GCe/TIV) and the ratio of contralateral GC volume to TIV (GC/TIV). The corresponding values of each dog's measurements were pooled for comparison. To assess the differences between the GC/TIV and GCe/TIV ratios within the study groups, a paired *t* test was performed for the dependent groups.

To evaluate the relationship between the GCe and GC, the general linear regression model was used.

The level of statistical significance was set at *P* < .05, and the analysis was performed by R in the Rstudio environment, by the tidyverse, ggplot2, and prcomp packages.

## 3 RESULTS

### 3.1 Study cohort

The study group comprised 32 dogs diagnosed with IE, including 9 female and 23 male dogs, weighing 5 to 46 kg (median: 19.5 kg). The IE group comprised 16 pure breeds (4 border collies, 3 Labrador retrievers, 2 golden retrievers, 2 beagles, 1 small Munsterlander, 1 German pointer, 1 Samoyed, and 1 Polish hound) and 16 mixed‐breed mesaticephalic dogs. The age at seizure onset ranged from 6 to 71 months (median, 24 months). The age at the time of MRI examination ranged from 8 to 72 months (median, 36 months). All dogs had a history of generalized seizures.

At the time of EEG examination, 20 dogs were not prescribed antiseizure medications (ASM). The prescriptions of antiepileptic drugs for the other dogs were: phenobarbital (*n* = 5); imepitoin (*n* = 2); phenobarbital and potassium bromide (*n* = 2); phenobarbital, potassium bromide, and levetiracetam (*n* = 1); phenobarbital, potassium bromide, gabapentin, and levetiracetam (*n* = 1); and phenobarbital, potassium bromide, and clonazepam (*n* = 1).

The control group included 13 dogs, 5 females and 8 males weighing 8.5 to 31.5 kg (median: 20 kg). Dogs in the control group underwent head MRI to exclude neurological causes. Seven patients were diagnosed with otitis externa (head shaking), 4 with degenerative disc disease (fibromuscular tremor), and 2 with pharyngitis (coughing). The control group comprised 2 pure breeds (1 border collie and 1 Italian shorthaired pointer) and 11 mixed‐breed mesaticephalic dogs. The age at the time of MRI examination ranged from 9 to 72 months (median: 32 months). There were no significant differences observed between the study and control groups in terms of age, weight, and sex. There were more purebred dogs in the study group than in the control group.

### 3.2 EEG results

**FIGURE 3:**
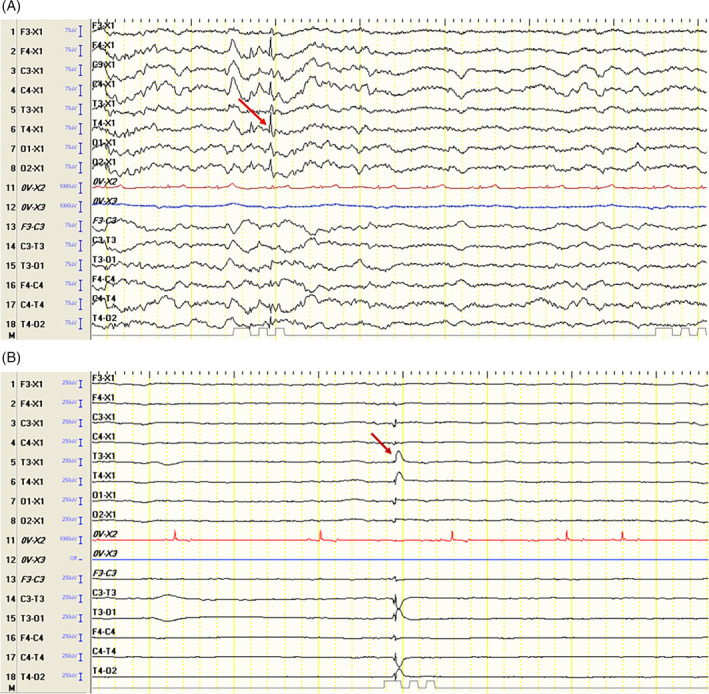
Electroencephalographic recordings show (A) spike activity from the T4 lead in a mixed‐breed dog and (B) a spike‐and‐wave complex from the T3 lead in a border collie.

In the study group, IEDs occurred in the left hemisphere in 18 dogs (56.3%) and the right hemisphere in 14 dogs (43.8%). Among the dogs with IEDs in the left hemisphere, all 18 had discharges at T3 (100%), 11 at C3 (61.1%), and 6 at F3 (33.3%). In contrast, among the dogs with IEDs in the right hemisphere, 14 dogs exhibited discharges at T4 (100%), 10 at C4 (71.4%), and 5 at F4 (35.7%; Figure 3A,B).

### 3.3 Volumetric measurements findings

**FIGURE 4:**
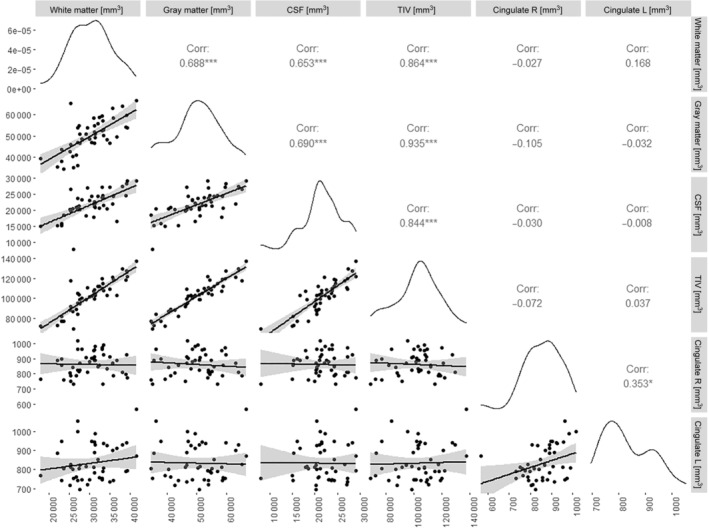
Correlogram of canine brain radiological variables. Asterisks indicate statistically significant relationships.

Because of the lack of data on semiautomated volumetric assessment of gray matter, white matter, and CSF in veterinary medicine, the first step was to collect these measurements as references alongside GC measurements and existing literature data.^36^ No significant differences were observed in white matter volume, gray matter volume, CSF volume, and TIV in either group. Significant correlations were observed among white matter volume, gray matter volume, CSF volume, and TIV. The correlations between imaging variables of the brain are shown in Figure 4.

In the group in which IEDs occurred in the left hemisphere, the GCe was smaller than the GC in 16 dogs (88.9%). In the group in which IEDs occurred in the right hemisphere, the GCe was smaller than the GC in all dogs.

**FIGURE 5:**
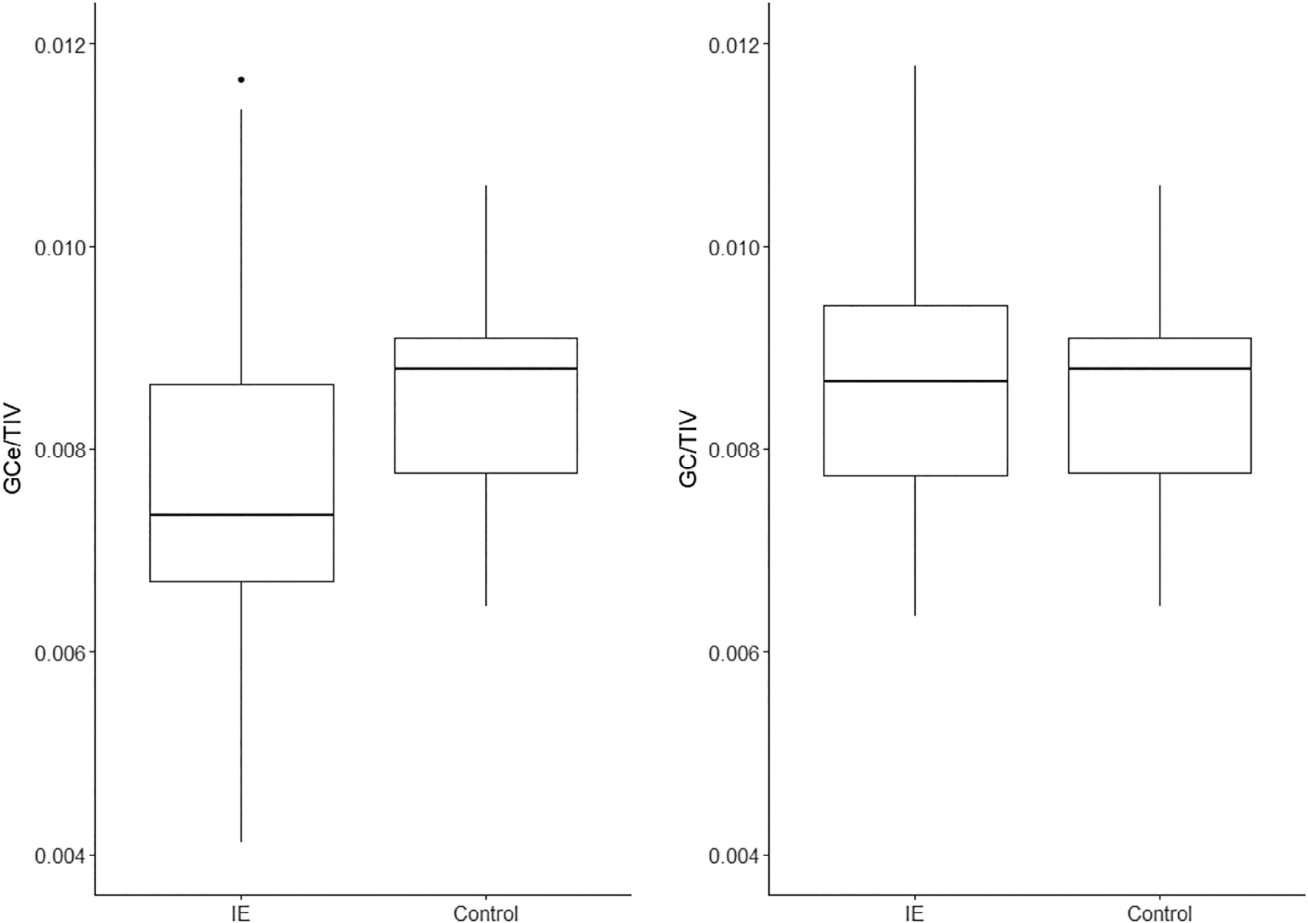
Comparison of the volume of the gyrus cingulatus/total intracranial volume ratio on the side of epileptogenic discharges (GCe/TIV) and contralateral gyrus (GC/TIV) to the average volume of the gyrus/total intracranial volume ratio in the control group.

**FIGURE 6:**
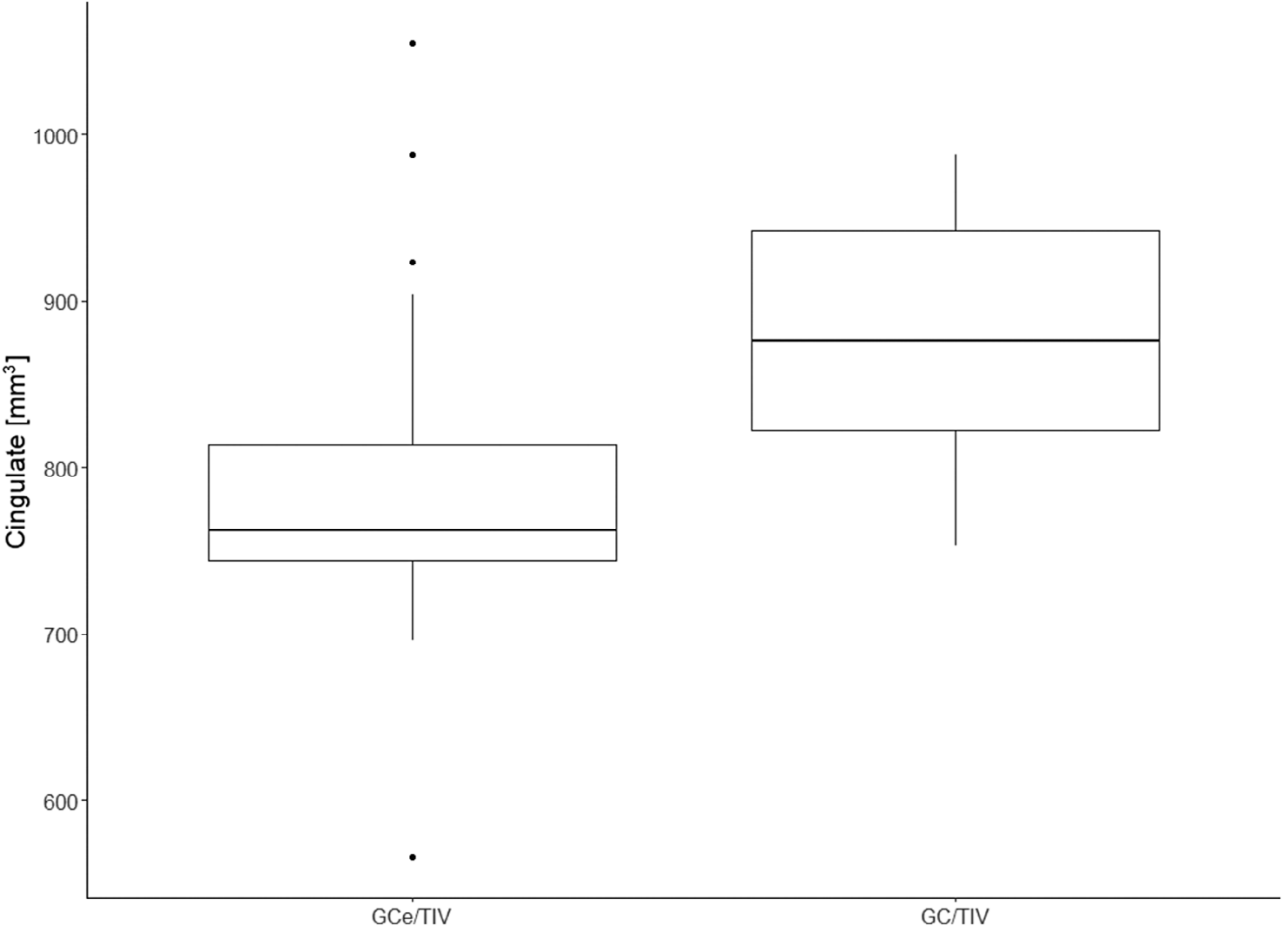
Comparison of the volume of the gyrus on the side of epileptogenic discharges/total intracranial volume ratio (GCe/TIV) and the contralateral gyrus/total intracranial volume ratio (GC/TIV) within the study group.

The study revealed a significant difference between the mean volume of the GCe in the IE group and the mean volume of the GC in the control group (*P* = .00044). However, there was no significant difference observed in the volume of the GC between the study group and the control group (*P* = .86). All variables are presented relative to the total brain volume (Figure 5). In dogs with IE, the GCe/TIV ratio was significantly lower than the GC/TIV ratio (*P* < .001; Figure 6).

To further explore the relationship between the GC/TIV and GCe/TIV ratios, a linear regression model was used. An outlier identified as having an impaired gyrus volume of <600 was excluded from the model.

**FIGURE 7:**
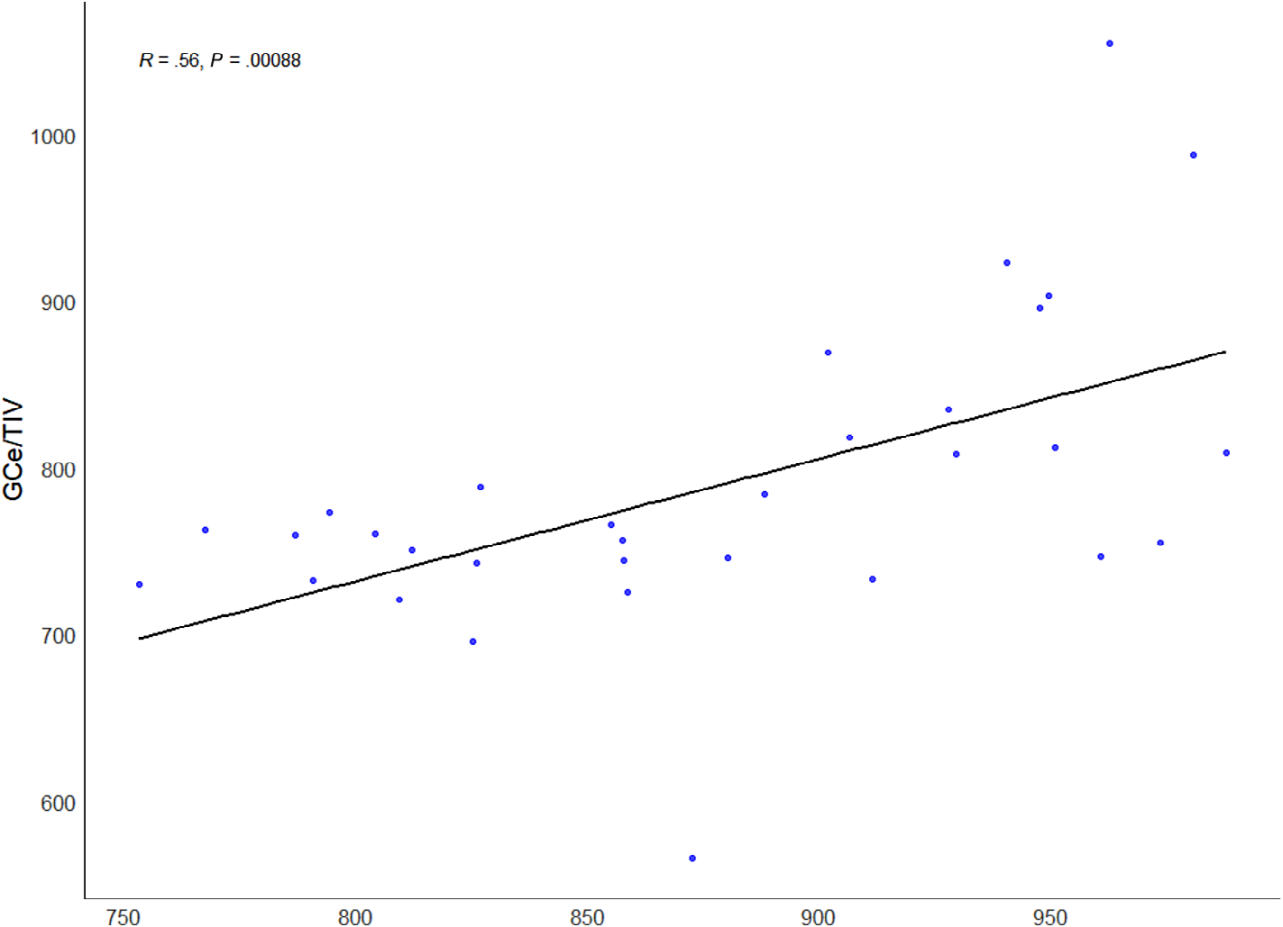
Correlation between the volume of the contralateral gyrus/total intracranial volume ratio (GC/TIV) and the volume of the gyrus on the side of epileptogenic discharges/total intracranial volume ratio (GCe/TIV; Pearson's correlation coefficient).

A significant correlation was observed between GC and GCe volumes in relation to TIV (*P* = .00153), with a correlation coefficient of .54 in the IE group. Regression analysis indicated that the GCe was typically approximately 30% smaller than the GC (regression coefficient: .68). The *R* ^2^ value for the presented regression was .28 (Figure 7).

## DISCUSSION

In this study, GCe was, on average, approximately 30% smaller than GC. However, there was no significant difference in GC between the study and control groups. This suggests similar structural changes observed in human medicine might occur in dogs with IE.

Human studies suggest that some subtle lesions can be missed by standard MRI protocols. Our findings emphasize the need for additional research to determine if changes in GC volume are primary lesions undetectable by standard MRI or, more likely, secondary lesions from epileptic discharges. This would highlight the importance of considering epilepsy as a network disease. Given the cingulate cortex's involvement in the limbic system,^3^ alterations in this structure could contribute to the behavioral comorbidities often seen in animals with epilepsy.^3^

Although the study revealed a reduction in GCe volume, it did not demonstrate a reduction in overall gray matter volume in dogs with epilepsy. This observation might stem from the fact that changes in gray matter volume relative to the cingulate cortex volume might have been too subtle to yield significant differences between the study and control groups. The most important differences in GCe and GC volumes were observed in a dog with drug‐resistant epilepsy (DRE). Additionally, in 2 animals, GC was larger than the GCe despite no visible MRI changes, suggesting possible peri‐ictal changes. This study confirms structural changes in the GC during IE, correlating with the side of IEDs. They underscore the need for novel imaging techniques like volumetry to assess structural brain changes in correlation with electroencephalographic evaluation.

In humans, VBM analyses of the GC reveal gray matter atrophy, mainly in the cranial GC and isthmus, in patients with IGE. Surface‐based comparisons revealed abnormalities, primarily in the caudal cingulate cortex.^17^ This atrophy in the GC correlates with the reduction in GCe volume observed in our study, although the changes in humans are more subtle. It is also worth noting that IGE in humans encompasses various types of epilepsy, such as childhood absence epilepsy (CAE), juvenile absence epilepsy (JAE), juvenile myoclonic epilepsy (JME), and epilepsy with GTCS alone (GTCA). However, the first 4 types are rarely recognized (or not established) in veterinary medicine. Our study focused on dogs suspected of having primary generalized tonic‐clonic seizures (equivalent to human GTCA in IGE).^17^ It is worth noting that the classification of epilepsy type was based on interviews with the owners and video recordings. Despite a thorough scheme that included questions about symptom onset, the appearance of epileptic episodes, and video recordings, we cannot exclude the possibility that some focal seizures were missed by the owners. This could change the classification from primary generalized tonic‐clonic seizures to focal epileptic seizures evolving into generalized seizures, which is an important limitation of the study. This point is crucial, as it could explain the atrophy of the GC on the side of IEDs observed in volumetric studies and the results of the EEG study. There is little agreement in the evaluation of focal epileptic seizures in dogs, supporting our hypothesis that some episodes might have gone unnoticed by owners.^37^

The cranial and caudal cingulate cortices are believed to play a role in the initiation or propagation of epileptogenic discharges in patients with IGE, potentially acting as primary drivers of the disease rather than consequences.^17^, ^38^ Human patients with IGE exhibit shorter commissural fiber bundles connecting the cranial cingulate cortex, suggesting the involvement of the bilateral cranial cingulate cortex in the pathophysiology of generalized tonic‐clonic seizures.^18^

Blood oxygen level‐dependent (BOLD) changes are noted primarily in the precuneus and caudal cingulate region starting approximately 10 s before generalized spike‐and‐wave (GSW) or polyspike‐and‐wave discharges in humans. At the onset of GSW discharges, BOLD enhancements are observed in the cranial GC, thalamus, and cerebellum. These studies collectively corroborate alterations in the cingulate cortex during IGE, which aligns with our findings in dogs.^19^

In veterinary medicine, brain imaging suggests that the limbic system, including the hippocampus and GC, is often affected in dogs with epilepsy. This could explain the high incidence of comorbid behavioral problems, such as anxiety and cognitive alterations.^3^, ^20^ Neuronal loss and gliosis in the limbic system are presenting, including the GC, amygdaloid nucleus, dorsal and ventral parts of the hippocampus, and dorsomedial nucleus of the thalamus, similar to postmortem findings in human patients with epilepsy originating in the limbic system.^39^, ^40^ In epileptic Shetland Sheepdogs that died of status epilepticus, astrocytosis and neurodegeneration predominate in the cingulate cortex and internal area of the frontal cortex.^41^ Our research supports structural changes in the GC during IE.

The largest difference between GC and GCe volumes was 217.99 mm^3^ in a dog with a gray matter volume of 59 537.2 mm^3^ and a TIV of 119 899.9 mm^3^. This difference was observed in a dog with suspected DRE receiving phenobarbital, potassium bromide, and clonazepam. In the other 2 animals with suspected DRE, the difference between GC and GCe volumes was 129.316 mm^3^, with a total gray matter volume of 50 711 mm^3^, and 113.168 mm^3^, with a total gray matter volume of 54 776.4 mm^3^.

In 2 dogs in the study group, GCe volume was higher than GC volume. In the first dog, GCe volume was 987.878 mm^3^, whereas GC volume was 980.627 mm^3^ (dog no. 17). This difference was within the range observed in the control group. The dog underwent MRI 6 months after the last observed generalized tonic‐clonic seizure. In the second dog, GC and GCe volumes were 962.388 and 1054.9 mm^3^, respectively (dog no. 12), a greater difference than observed in the control group. This animal underwent an MRI 1 month after the last observed generalized tonic‐clonic seizure.

The absence of lesions detected by the standard MRI protocol and based on volumetric analysis does not rule out the presence of lesions. Given the available data and literature, it remains challenging to ascertain whether these lesions are secondary to epileptic discharges or if they represent primary lesions, particularly considering that all dogs in the study group had experienced epileptic seizures before undergoing an MRI examination. Although most animals in the study group exhibited a reduced volume of GCe relative to GC, in 2 cases, the ratio was reversed. Notably, no visible structural changes, including peri‐ictal changes described in veterinary medicine, are observed in these cases.^24^ Nevertheless, we cannot exclude the possibility of subtle changes, such as vasogenic or cytotoxic edema described as peri‐ictal changes, because postattack lesions can persist up to 16 weeks after a seizure, varying in intensity depending on the time since the last epileptic seizure.^42^

It is plausible that these changes were too subtle to be discerned by the standard MRI protocol, whereas the volume of GCe in volumetric analysis still increased. In dogs with significantly smaller GCe volumes than GC volumes, both primary lesions and those secondary to epileptic discharges must be considered. In both human and veterinary medicine, peri‐ictal and postictal changes are well described.^24^, ^42^ Studies by means of techniques based on nerve tissue diffusion evaluation have distinguished several stages of postacute lesions depending on the apparent diffusion coefficient: (1) initial regional hyperperfusion, (2) vasogenic edema formation, (3) cytotoxic edema formation, and (4) progressive neuronal loss or gliosis.^24^ Given that gliosis and its consequent cell shrinkage and neuronal loss are linked to a reduction in neural tissue volume, these changes could be responsible for the observed decrease in the volume of the GC in our study.

Based on these results, the presence of primary lesions should also be considered, especially in animals with drug‐resistant epilepsy. Approximately 30% of focal epileptogenic lesions are missed on standard MRI in human medicine, necessitating advanced neuroimaging modalities.^11^ This suggests that dogs diagnosed with IE might have structural lesions undetectable by standard MRI, potentially classifying them as having structural epilepsy.

Structural disorders to consider, based on human and veterinary medicine, include congenital cortical malformations like focal cortical dysplasia (FCD). Cortical dysgenesis (CD) encompasses a wide spectrum of brain anomalies involving abnormal development of the cerebral cortex and is strongly associated with epilepsy, often drug‐resistant.^43^ During FCD, foci of increased gray matter thickness or atrophy are observed. Epileptogenic zones in brain structures with abnormal volume were found in 87.5% of patients, specifically in 71.4% of patients with atrophic volume. This suggests that FCD lesions are more likely to occur in regions with an atrophic volume than in those with a hypertrophic volume.^44^ In humans, FCD has been found in the GC; however, it was associated with GC epilepsy rather than IGE.^45^ There are few reported cases of dogs with CD, as it is rarely diagnosed in companion animals.^46^, ^47^ Notably, in this case, brain MRI revealed no abnormalities on 3‐mm transverse sections.^47^

The primary question is whether dogs currently classified as having IE actually have subtle lesions that are not visible by the standard MRI protocol, which would reclassify them as having structural epilepsy. This is particularly relevant for dogs with DRE, who account for up to 30% of dogs with IE. Because the animals did not undergo follow‐up MRI, the presence of progressive lesions such as inflammatory, neoplastic, or neurodegenerative changes cannot be fully excluded. However, given the age at seizure onset, the nature of the seizures, and the results of tests performed according to the IVETF‐recommended protocol, the occurrence of such lesions is unlikely in our opinion.

Recent intensive research on IE in animals has led to the successful application of some advanced diagnostic techniques in dogs with epilepsy, with several methods now implemented in veterinary medicine.^4^, ^5^, ^16^, ^48^, ^49^, ^50^, ^51^, ^52^, ^53^ Our study aimed to establish correlations between the semiautomated volumetry of the GC and the localization of IEDs during the course of IE.

Volumetric analysis is a common diagnostic method in advanced neuroimaging. In human medicine, it is fully automated and aids in diagnosing many neurological diseases, including epilepsy.^14^, ^54^ Before the development of canine brain atlases, volumetry could only be performed manually, which was less sensitive than automated methods.^32^ The use of semiautomatic volumetrics is advantageous because it eliminates human error in marking regions of interest. Additionally, this method could be very helpful in locating focus epilepticus, thereby contributing to the advancements in epilepsy diagnostics and treatment.

EEG, an important diagnostic tool in human medicine, might also aid in diagnosing epilepsy in dogs by confirming abnormal brain activity through visible EDs or IEDs and their locations. Combining EEG with MR brain volumetry can enhance the analysis of the brain during epilepsy, including the precise localization of epileptogenic sites. Based on our study, we suspect that dogs with IE and generalized tonic‐clonic epileptic seizures have subtle structural changes in the GCe, which are not detectable by standard MRI protocols. In our opinion, the reduction of GCe volume results from cortical atrophy caused by chronic repetitive damage to this area from the epileptic seizure propagation, leading to structural damage. However, the study results must be interpreted carefully owing to its limitations.

The main limitation of this study is that all animals experienced seizures before the MRI examination. Consequently, we cannot determine whether the observed reduction in GC volume is secondary to epileptic attacks or a primary change. However, in similar human studies, all patients who qualified for the research also had epileptic seizures before MRI.

Another limitation is the use of semiautomatic brain volumetry. Fully automated volumetry would eliminate human error, but because of the diversity of skull types within the *Canis familiaris* species, even when including only mesaticephalic dogs, such volumetry is currently impossible.

The research was performed by a 1.5 T MRI. Studies in human medicine suggest a 5% increase in diagnostic efficiency for FCD when using a 3 T MRI instead of a 1.5 T MRI.^55^ Therefore, subtle structural changes might have been overlooked in the present study. Despite this, according to the IVETF protocol, 1.5 T is considered diagnostic.^16^, ^34^ Furthermore, technical constraints related to using a 1.5 T MRI, combined with the size of the dog's brain, prevented differentiation between the cranial and caudal portions of the GC, which is feasible in human studies.^17^, ^18^, ^19^

The age of seizure onset in dogs diagnosed with IE according to the IVETF criteria ranges from 6 months to 6 years, creating a significant variation in the age of affected patients, which was noticeable in our study.^9^, ^16^ We believe that the data obtained in this study are valuable as they represent a cross‐section of a diverse group of dogs with IE.

Additionally, some animals underwent ASM during EEG examination, making the group heterogeneous. This might have resulted in fewer epileptic discharges in some animals during the study.^56^ Therefore, it was impossible to establish an exact correlation between the severity of epileptic seizures and changes in the GC volume.

Another important issue is the limitations of EEG. In our study, a 30‐minute recording protocol was used. In humans, this type of examination provides a 50% seizure detection rate, whereas prolonged EEG monitoring increases the yield to 70% to 80%. Additionally, a repeated second EEG examination further enhances the yield to 90%.^57^, ^58^, ^59^ Therefore, we cannot exclude the possibility that longer or repeated EEG studies might have detected discharges in additional leads. Another problem is the lack of standardization for EEG usage and technique, unlike the established protocols in human epileptology.^12^

Because all examined animals were alive, it was impossible to conduct a histopathological examination to determine the nature of the changes.

The study revealed that the GCe is, on average, approximately 30% smaller than the GC relative to TIV. Our research suggests that the GC possibly plays a role in the initiation or propagation of EDs in patients with IGE; however, further investigation is needed to test this hypothesis. Drawing from research in human medicine indicating that approximately 30% of focal epileptogenic lesions might be overlooked on standard MRI,^11^ there is a pressing need for new diagnostic methods in both human and veterinary medicine. Moreover, given the perception of epilepsy as a network disease and the frequent occurrence of comorbid behavioral disorders in animals with IE, a comprehensive structural analysis of the limbic system, including the GC, is imperative for understanding the etiology and consequences of this disorder.^3^, ^20^, ^21^ The study confirms the presence of structural changes in the GC during epileptic seizures, contributing to a deeper understanding of brain structure in the context of epileptic seizures. Furthermore, it might facilitate improved classification of epileptic patients and potentially influence the direction of research in epileptology.

## CONFLICT OF INTEREST DECLARATION

The authors declare that they have no competing interests.

## OFF‐LABEL ANTIMICROBIAL DECLARATION

Authors declare no off‐label use of antimicrobials.

## INSTITUTIONAL ANIMAL CARE AND USE COMMITTEE (IACUC) OR OTHER APPROVAL DECLARATION

According to Polish law, ethical committee approval was not mandated for this study (Experiments on Animals Act from January 15, 2015, *Journal of Laws of the Republic of Poland* from 2015, item. 266).

## HUMAN ETHICS APPROVAL DECLARATION

Authors declare human ethics approval was not needed for this study.
